# Integrin-mediated function of Rab GTPases in cancer progression

**DOI:** 10.1186/1476-4598-9-312

**Published:** 2010-12-09

**Authors:** Dhatchayini Subramani, Suresh K Alahari

**Affiliations:** 1 Department of Biochemistry, Stanley S. Scott Cancer Center, LSU School of Medicine 1901 Perdido Street, New Orleans, LA 70112, USA

## Abstract

The RAS (rat sarcoma) superfamily of small GTPases is broadly subdivided into five groups: Ras, Rho, Rab, Ran, and Arf. Rab family proteins are important in regulating signal transduction and cellular processes such as differentiation, proliferation, vesicle transport, nuclear assembly, and cytoskeleton formation. However, some Rab proteins have been reported to be necessary for the adhesion and migration of cancer cells. Although Ras and Rho family members have been strongly implicated in cancer progression, knowledge of Rabs action in this regard is limited. Some reports have also linked Rab GTPases with cancer cell migration and invasiveness. This review discusses the implications of the involvement of Rabs in malignant transformation and cancer therapy through integrin-mediated signaling events, with particular emphasis on breast cancer.

## Introduction

**Figure 1:**
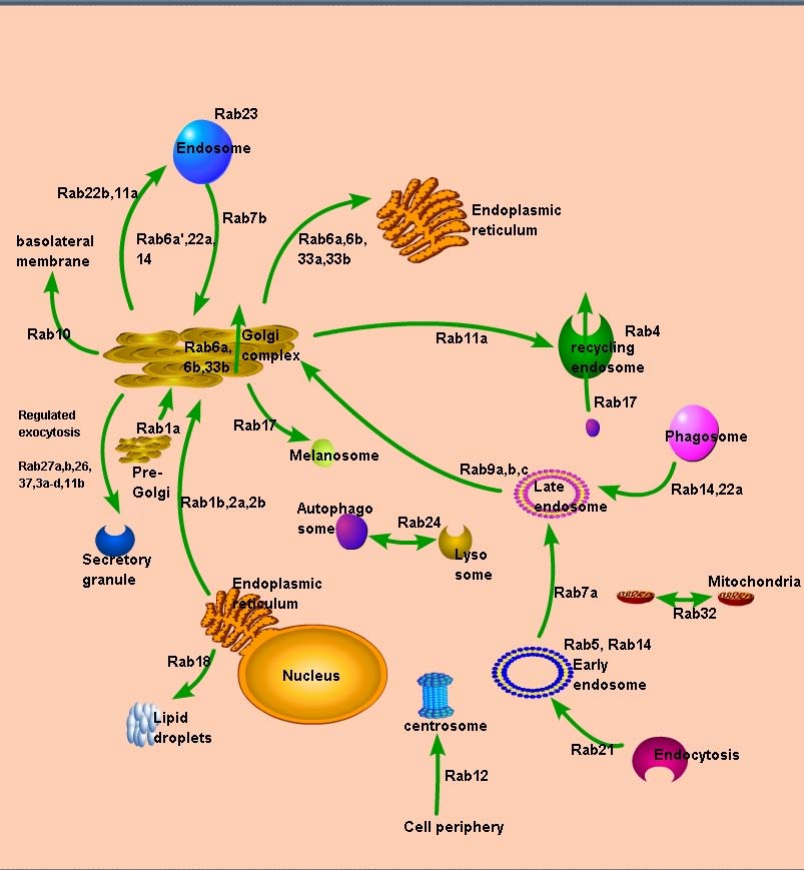
**Rab GTPases are involved in vesicle transport and trafficking**. Some examples of Rab functions are shown. Rab 1a is localized pre-Golgi intermediates, and Rab 1b, 2a and 2b, which are localized in the ER, are important in transport from the ER to Golgi. Rab6a, 6b, and 33b are involved in intra-Golgi transport, as well as transport from the Golgi apparatus to the ER. Rab 11a and Rab17 are involved in transport through apical recycling endosomes. Rab14 and 22a contribute to transport from phagosomes to late endosomes. Rab9a, 9b, and 9c are involved in late endosome to Golgi transport. Rab7b participates in transport from endosomes to the TGN, while Rab12 is involved in transport from the cell periphery to the centrosome. Rab 5 and 14 are localized in early endosome and Rab 4 is localized in recycled enodosmes. Rab18 is involved in transport from ER to lipid droplets; Rab10, from Golgi to the basolateral membrane; Rab 26, 27a, 27b, 37, 11b and 3a-d, from Golgi to secretory granules; Rab22b and Rab11a, from Golgi to endosomes; and Rab6a', 22a and 14, from endosomes to Golgi. Rab 32 is localized in mitochondria and regulates mitochondrial dynamics.

Ras-associated binding (Rab)-GTPases are members of the Ras family of small GTPases. They are abnormally expressed in different cancer tissues. Almost 70 Rabs and Rab-like proteins are encoded by the human genome. Some of these are tissue-specific; most of them are ubiquitously expressed (Figure 1). Rabs participate in receptor cargo collection during transport vesicle formation and enable motor proteins to interact with membranes to drive vesicle motility, mediating the complex events of accurate docking and fusion of transport vesicles with their targets. Protein-protein interactions are important for localization of Rabs in mammalian cells [1].

Rab GTPases mediate vesicle formation and vesicle movement along with actin and tubulin, and membrane fusion [2]. This process is involved in cell migration with lamellipodial formation. The Rab subfamily terminates in a set of cysteine-containing C-terminal motifs that allow modification of Rab GTPases to various membrane locations by geranyl-geranyl transferase II [3]. The Rab regulators promote a switch between the GDP-bound off-state and a GTP-bound on-state. The switch between GDP and GTP bound forms of Rab GTPases occurs through major conformational changes in two variable regions, Switch I and Switch II. GDP to GTP conversion is catalyzed by guanine nucleotide exchange factors (GEFs), which recognize particular residues in the switch regions and cause GDP release. The switch from GTP to the GDP bound form occurs through GTP hydrolysis, which is catalyzed by GTPase-activating proteins (GAPs) [4,5]. At times, Rab GTPases exist in GDP bound forms. This is caused by Rab GTPase dissociation inhibitor (GDI), which prevents the release of GDP from Rab. For specific recognition, a vesicle and its target are coded by a unique compliment of Rab GTPases [6]. Thus, it is important to understand the intracellular signaling of Rab proteins in various cells to find new molecular approaches to cancer biology. We briefly summarize the functions of all major Rab proteins in vesicular trafficking; describe their function in endosomes and the importance of Rabs in cancer biology; and discuss the importance of integrins in the regulation of Rab functions.

### Localization and function of Rab GTPases

A total of 51 Rab and Rab-related proteins have been functionally characterized. As shown in Figure 1, several Rabs are localized in endoplasmic reticulum (ER) and involved in transport from ER to Golgi. Some Rabs are localized in endosomes, clathrin-coated vesicles, plasma membrane, membrane ruffles, and secretory granules (Figure 1). These Rabs have a wide variety of functions that include, but not limited to, trafficking between Golgi and endosomes, phagocytosis, the assembly of adherens junctions, trafficking of sonic hedgehog signaling components, mitochondrial dynamics, and neuroendocrine secretion (Figure 1, Additional File 1). Here we briefly discuss the function of Rab and Rab-related proteins in membrane trafficking.

Rab GTPases are involved in regulating membrane trafficking between organelles through the recruitment of effector proteins. Immunodeficiencies, cancer, and neurological disorders are associated with functional impairments of Rab signaling pathways. Many important reports have linked Rab GTPases to cancer cell migration and invasiveness. The two Rab GTPases, Rab5 and Rab21, are associated with subunits of alpha-integrin, altering their endosomal traffic and subcellular localizations.

The regulated trafficking of vesicles to the cell surface is fundamental to all cells. Rab proteins are key mediators of vesicle transport. Rab GTPases are important in delivering cargos to correct destinations and control vesicle budding, as well as the uncoating and motility of vesicles. Rab GTPases, with the help of their effectors ensure the spatio-temporal regulation of vesicle traffic. Furthermore, temporal and spatial regulation of membrane trafficking events is important for membrane identity and cell polarity.

Although the exact mechanism of targeting different Rab GTPases is not clear, membrane-bound GDI displacement factors have an important function in recognizing specific Rab GDI complexes and allowing GDI release. This release helps in the association between geranyl-geranylated Rab GTPases and the appropriate membranes. An important step in intracellular trafficking is cargo selection into specific transport vesicles. Membrane curvature, lipid composition, and Rab GTPases determine the specific coat recruitment to particular intracellular membranes. Rab 9 and Rab 5 are two good examples of Rab GTPases that are involved in cargo selection [7,8]. Rab 9 is involved in cargo specific-coat assembly for the recycling of mannose phosphate receptors from late endosomes to the trans-Golgi network. Rab 5 is important in cargo sequestration, and is crucial for the assembly of clathrin-coated pits and clathrin-mediated endocytosis of transferring receptors.

Some Rab GTPases control several crucial events in vesicle budding from donor membranes, including coat assembly, cargo selection, and physical budding. Vesicle coat complexes are important for cargo sequestration and membrane budding; however, the coat complexes interfere with membrane fusion and thus need to be shed before association with an acceptor membrane can occur. For example, Rab5, the GEF and GAP of Rab 5 are involved in uncoating events [9]. Actin filaments and microtubules regulate the direction and accuracy of vesicle delivery. Rab GTPases proofread the specific attachment of motors to vesicles. For example, Rab 27A recruits melanophilin to connect melanosomes to myosin Va [10]. Rab GTPases also mediate vesicle docking or fusion. Some Rab GTPases enhance fusion of TGN-derived vesicles with the plasma membrane. Rab5 is required for homotypic early endosome fusion and the fusion of endocytic vesicles to endosomes [11].

Several reports have indicated that Rab proteins can bind to various proteins to facilitate membrane transport [reviewed in[12]]. Active Rab proteins are also required for vesicle budding. Membrane budding to form vesicles involves interaction between several proteins. First, Rab proteins are recruited and activated at the donor membrane, where they are important in vesicle budding. Second, Rab proteins facilitate transport along the cytoskeleton. Finally, they are involved in docking and fusion. Thus, Rab proteins control several events during vesicular transport [12].

### Involvement of Rab GTPases in different Cancers

**Figure 2:**
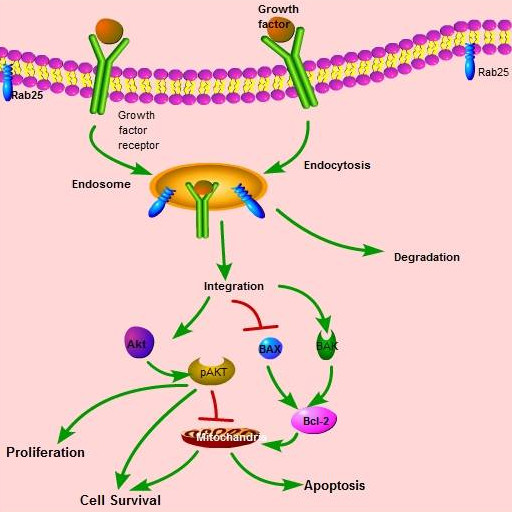
**Role of Rab25 in cancer**. Activated Rab 25 and other growth factor receptors are located near the cell surface. Rab25 regulates receptor internalization, vesicle formation, and trafficking with specific cellular components. Rab25 also activates AKT, which increases cell proliferation and survival. Rab25 integration leads to down-regulation of BAX and BAK to activate the Bcl2 pathway, which affects apoptosis and cell survival.

Some Rab GTPases regulate tumorigenesis via trafficking mediated events and some function in a trafficking independent way (Figure 2). In this section, we will discuss both of them.

#### Trafficking-dependent tumorigenic regulators

Although Rab proteins are involved in intracellular vesicle transport, they also have a major role in cancer progression. Here we mainly focus on Rab proteins, which are especially important in this respect. In particular, the role of Rab25 in cancer progression has been studied at great length.

##### The role of RAB 25 in cancer

Rab25, a 28-kDa protein also referred to as Rab11c, is involved in cell proliferation and protection from apoptosis. It has high levels of expression in ovarian and breast cancers, in which it has been shown to have a key function in both *in vitro *and *in vivo *cancer progression [13-15]. Increased expression of Rab25 has also been noted in prostate cancer [16] and transitional cell carcinoma of the bladder [17], indicating that it has a pathological role in tumor progression in several epithelial lineages. Reports indicate that Rab25 is involved in tumor progression by regulating the localization of integrin-recycling vesicles to enhance tumor invasion (Figure 2).

Rab25 has been shown to decrease apoptosis, as well as to increase both the proliferation and aggressiveness of ovarian and breast cancer [18]. Regulation of cell survival is mediated by antiapoptotic molecules such as Bcl-2, BAX, BAK, and phosphoinositide-3-kinase [19,20]. Forced expression of Rab25 in ovarian cancer cells decreases levels of BAX and BAK [21] and increases AKT phosphorylation, which in turn activates the phosphoinositide-3-kinase pathway. Down-regulation of Rab25 reverses apoptotic signaling through BAK and BAX protein signaling, as well as AKT activity. The Bcl-2 and phosphoinositide-3-kinase pathways are enhanced by Rab25-mediated signaling for cell survival (Figure 2)[18].

Goldenring et al. [22] demonstrated that Rab 25 expression is increased in colon cancer cells. Mills and colleagues [14] showed that 1q22 amplicon containing Rab25 is amplified in 50% of ovarian cancers. Further in vivo studies confirmed that expression of Rab25 increases ovarian tumor growth. In addition, chromosome 1q is gained in 50% of breast cancer patients. Cheng and colleagues[23] showed high expression of Rab25 in 92% of ER-positive samples and loss of expression in 83% ER-negative and PR-negative samples of basal and triple-negative tumors. These findings suggest that Rab25 may have different functions in different subtypes of breast cancer. In addition, Rab 25 is overexpressed in Wilms tumor, early-stage transitional cell carcinoma of the bladder, and hepatocellular carcinoma.

In contrast, Rab25 has also been shown to function as a tumor suppressor; its expression is lost in hormonally insensitive breast tumors [24]. Another recent study indicated that Rab25 may function as a tumor suppressor in intestinal cells. This study demonstrates that loss of Rab25 leads to increased tumorigenesis through alteration in the regulation of protein trafficking to the cell surface. These findings suggest that Rab 25 functions as a tumor suppressor in the intestinal mucosa and leading to human colorectal adenocarcinoma [25]. Rab25 knockdown by shRNA shows less proliferation, more apoptosis, and decreased ovarian tumor growth both *in vitro *and *in vivo *[13]. Studies on human breast cancer tissues have shown that loss of Rab25 expression occurs through a mutation in locus 1q22-23. Thus, Rab25 could be used as a biological marker of breast cancer [23].

Rab5 regulates the fusogenic properties of early endosomes through GTP-dependent recruitment and activation of effector proteins. Defective Rab5 causes the formation of enlarged multivesicular endosomes with many intraluminal vesicles; endosomes with early and late endocytic markers are frequently observed in the presence of defective Rab5. Inactive Rab5 is defective in sorting EGF receptor and transferrin receptor [26]. Cdc42 interacting protein-4 (CIP-4) localizes to vesicles with EGFR and the small GTPase Rab5. In giant endosomes, expression of constitutively active Rab5 leads to the accumulation of CIP-4 and related adaptor Toca-1. Downregulation of CIP-4 expression in A431 epidermoid carcinoma cells causes elevation of EGFR but, surprisingly, does not affect surface expression of EGFR [27]. Thus, understanding the function of Rab5 in EGFR signaling will be of great interest.

Rab5 guanine nucleotide exchange factor Rin1, which is activated by Ras, is important in growth-factor receptor trafficking in fibroblasts and endocytosis. Rin1 is highly expressed in the lung adenocarcinoma cell lines Hop62, H650, HCC4006, HCC827, EKVX, HCC2935, and A549. Cell proliferation is reduced by depletion of Rin1 in A549 cells, and is correlated with a decrease in EGFR signaling. Thus, Tomshine et al proposed not only that EGFR-mediated signaling essentially requires proper internalization and endocytic trafficking, but that upregulation of Rab5 guanine nucleotide exchange factor Rin1 may be involved in the proliferative pathway in A549 cells [28].

Rab11 co-localizes with insulin-containing granules in the mouse insulin-secreting cell line MIN6. Overexpression of Rab11B inhibits insulin secretion, indicating its involvement in regulating insulin secretion. Rip11, an effector of Rab11, is a substrate for PKA, which regulates exocytosis in pancreatic β-cells [29]. Rip11, which participates in insulin granule exocytosis, also has been reported to regulate the recycling of internalized cell-surface proteins from the early endosome through a trans-Golgi network. Expression of Rip11 is higher in ductal carcinoma in situ (DCIS) than it is in normal breast epithelium. Rab11a influences EGFR recycling, enhances proliferation, and prevents motility of an immortal breast cell line (MCF 10A), which is consistent with the phenotype of DCIS [30]. Rab11a and Rab11-family interacting protein 2 (FIP2) are involved in the regulation of plasma membrane recycling in epithelial cells. The phosphorylation of Rab11-FIP2 on serine 227 by MARK2 is required for epithelial cell polarity [31]. EGFR, which initiates many biochemical pathways to potentiate physiological responses, also has an important part in cancer progression. The recycling of EGFR and its signaling pathway is regulated by the endocytic pathway [32]. ADP-ribosylation factor 6 (Arf6), a regulator of cytoskeletal dynamics during cell movement, binds with protein FIP3, which interacts with Arf6 and Rab11. Both Arf6 and Rab11 appear to be involved in regulating cell motility. MDA-MB-231 breast cancer cells require FIP3 for motility. FIP3 also is involved in regulating the localization of Arf6 in MDA-MB-231 breast cancer cells and polarization of Rac1 [33].

The Rab-coupling protein RCP (RAB11FIP1), located on chromosome 8p11-12, encodes for a protein with Ras-activating function. RCP is frequently amplified in breast cancer. An in-vitro study with normal human mammary epithelial cells (MCF10A) has shown that overexpression of RCP not only increases loss of contact inhibition, growth-factor independence, and anchorage-independent growth, but also promotes Ras activation and ERK phosphorylation. Consistent with this, knockdown of RCP inhibits tumor formation and metastasis in a xenograft model [34]. Rab11 and the Eps15 homology domain 1 (EAD1) regulate the exit of internalized molecules from endocytic recycling to the plasma membrane. Thus, coordination between Rab11 and EAD proteins occurs in the mediation of endocytic recycling [35].

Rab27A is involved in exocytosis of endocrine cells and is associated with the invasive and metastatic potential of breast cancer, promoting the secretion of insulin-like growth factor-II (IGF-II). The rate of secretion controls the expression of vascular endothelial growth factor, matrix metalloproteinase-9 (MMP-9), cathepsin D, cyclin D1, p16, and urokinase-type plasminogen activator [36]. Mutations in Rab27A lead to an autosomal-recessive immunodeficiency called Griscelli syndrome type 2. Clinically, this syndrome is characterized by partial albinism and hemophagocytic lymphohistocytosis. Understanding the mechanisms of Rab27A mutations may be helpful in early diagnosis and treatment [37]. An elegant study recently demonstrated that Rab 27B controls vesicle exocytosis and releases important growth regulators into the tumor microenvironment, leading to regulation of invasive growth and metastasis in ER-positive human breast tumors [38]. This study also suggested that increased expression of Rab27 is associated with poor prognosis in humans [38] suggesting Rab 27b may function like an oncogene.

Microarray analysis data indicate that Rab35 is highly expressed in ovarian cancer. It also has been shown that androgens may affect the expression of oncogenic GTPases in ovarian cancer [29]. Rab35 is involved in T-cell receptor and oocyte yolk protein recycling and cytokinesis. Chua et al. (2010) have shown that Rab35 causes actin to reconcile protrusion through either Rho GTPases or effector fascin of actin bundling protein [39].

Rab4 and Rab14 are also involved in vesicle trafficking and associate with the C-terminal domain of P-glycoprotein (P-gp), which induces signaling for a multi-drug-resistance (MDR) mechanism in tumors. Overexpression of Rab4, but not Rab14, decreases P-gp on the cell surface of K562ADR cells, reducing the MDR phenotype by elevating the intracellular addition of daunomycin [40]. Accordingly, modulation of the spatial and temporal distribution of P-gp may be a good therapeutic strategy for suppressing the MDR phenotype.

#### Trafficking-independent tumorigenic regulators

Rab 23 binds to PSF (Polypyrimidine tract-binding protein (PTB)- associated splicing factor), a tumor suppressor; PSF and VL30-1 RNA, a mouse retroelement noncoding RNA, are involved in reversible regulation of proto-oncogene transcription, cell proliferation, and tumorigenesis in a mouse model. PSF represses transcription by binding with the proto-oncogene Rab23; the VL30-1 RNA binds with PSF to release it from Rab23 for activating transcription. PSF and its binding RNAs have a major function in reversible regulation of mammalian cell proliferation and tumorigenensis. Indeed, alteration in the expression of PSF and its binding RNAs may have potential as a therapeutic strategy against cancer [41]. Human RAB37 (hRAB37) acts as a metastasis-related tumor suppressor gene (TSG) in lung cancer. Methylation in the promoter is the main mechanism for down-regulation of hRAB37 [42]. Thus Rab23 and Rab 37 regulate tumorigenesis through the mechanisms other than trafficking.

##### Rab GTPases and Integrins

Integrins are members of a glycoprotein family that form heterodimeric receptors involved in interaction between the cell and the surrounding extracellular matrix (ECM). Nineteen alpha subunits and eight beta subunits are paired to form 25 different integrins. Integrins control several biological processes, including cell adhesion, cell migration, and the progression of cancer and some other diseases [43,44]. Integrins are also involved in transducing signals from outside the cells to inside and from inside to outside the cells. The ligand binding abilities of integrins are determined by the α/β subunit heterodimerization. The ligands for integrins are several extracellular matrix proteins including collagen, laminin, vitronectin and fibronectin. Some integrins such as α5β1 interact with a single ECM protein, fibronectin, however, usually integrins recognize several distinct matrix proteins [45].

The cytoplasmic domains of integrins provide a key connection between the ECM and intracellular proteins and signaling mechanisms [43]. Although both alpha and beta subunits make important contributions to various aspects of integrin functions including signal transduction, cell motility, cell adhesion and integrin activation, the beta cytoplasmic domains are critical for recruitment of integrins to focal contacts (the specialized structures at cell-matrix junctions). Integrins can also respond to signals generated inside the cells that may impact on the cytoplasmic domain, which is often referred to as inside-out signaling. For example, truncation of some integrin cytoplasmic domains prevents integrin activation [46].

#### Integrins and Cell Adhesion

It is apparently clear that integrins can directly activate intracellular signaling processes without the requirement of growth factors. Integrin mediated adhesion activates tyrosine phosphorylation of focal adhesion kinase (FAK) [47,48], and FAK in turn binds to other signaling and structural proteins including c-Src, PI3K, paxillin and p130CAS. Furthermore, adhesion triggered activation of ERK has been shown. In this scenario, upon cell adhesion, FAK is recruited to focal contacts, phosphorylated at Y397 and Src is recruited, which then phosphorylates at other sites. FAK then binds to Grb2 and Grb2/SOS complex activates Ras-Raf-MEK-ERK cascade [49]. In addition, cell adhesion (integrin engagement) activates Rho GTPases, which play an important role in cytoskeletal regulation and cell migration [44].

#### Integrins and Cell Migration

Cell migration involves very well orchestrated events that allow cells to attach to substratum and detach from it, to protrude projections and directionally advance. Cell motility consists of four major events including extension of the lamellipod, formation of new focal adhesion at the leading edge, breaking of adhesions at the rear end and translocation of the cell mass [43]. Almost all of the events of cell migration involve adhesion receptors especially integrins. As described above FAK associates with focal adhesions and gets phosphorylated upon integrin mediated cell attachment to the ECM. In several cell types, FAK has been shown to promote cell migration [50]. FAK directly binds to the adaptor protein that interacts with activated p130 CAS. This complex promotes cell migration and invasion of cancer cells through Rac signaling cascade. Also ERK promotes cell migration by regulation of actin-myosin assembly and cell contraction. Thus, both ERK activation and CAS/CRK complexes coordinately regulate cell migration (reviewed in [43].

#### Integrins and Endocytosis

The endocytosis and exocytosis cycle of integrins is a key regulator of cell adhesion, migration, and the maintenance of cell polarity. Several recent reports indicate that members of the Rab family regulate the internalization of integrin and its recycling back to the cell membrane. The original concept has been that exocytosis at their leading edge helps cells move by producing new adhesion receptors, which are internalized at the rear end of the cells. The cytoplasmic domains of integrins have a major function in the trafficking of integrins. A membrane proximal YRRF motif in beta integrins and a GFFKR motif in alpha integrin are important in regulating membrane traffic [51,52].

The endocytosis of integrins occurs through clathrin-dependent and -independent pathways. Recently, it has become clear that integrins are internalized at focal adhesions near the leading edge of cells [53]. Thus, integrin trafficking seems to be important in maintaining migrational polarity and localizing integrin signaling at the front of cells. Several Rab GTPases control integrin recycling. Rab 4 effector protein, Rab 5, Rab 21, Rab 11, Rab 25, Rab 4A, Rab 8A, and Rab 11FIP1 are of great interest in this context.

Rab 4 effector protein (Rab IP4) is involved in recycling αvβ3 integrins. Inhibition of Rab IP4 function blocks integrin recycling, leading to inhibition of cell adhesion and cell spreading [54]. The integrin translocation from the trailing edge toward the advancing lamellipodium seems to be important for the speed and direction of cell migration. In migrating cells, integrin distribution is polarized in the leading edge; endosomal transport is a major factor in this translocation. Consistent with this hypothesis, suppression of Rab IP4 does not allow αvβ3 integrin to localize in the leading edge of migrating cells. Several reports indicate that integrin-containing vesicles move from the rear end of cells to the lamellipodia and that dysregulation of integrin function leads to the pathogenesis of cancer. Integrin engagement promotes proliferation and the apoptotic resistance of cancer cells, and regulates cell migration and invasion during metastasis.

Rab5 and Rab 21 regulate endocytosis and the recycling of beta integrins. Rab5 family GTPases regulates endocytosis through the movement of endocytic vesicles along microtubules. Caspase-8 has a major part in controlling cellular responses to promote apoptosis, proliferation, and cell migration. Protein caspase-8-mediated signaling from integrins is regulated by Rab5. Integrin-mediated activation of Rab5 enhances colocalization and coprecipitation of integrins with caspase-8. Rab5-mediated internalization and recycling of β1 integrins are increased by caspase-8. Knockdown of Rab5 prevents integrin signaling from activating Rac to promote cell migration and the apoptotic signaling pathway. Caspase-8-driven cell migration critically requires Rab 5. Thus, Rab 5 is an essential integrator of caspase-8-mediated signal transduction to regulate cell survival and migration [55].

Rab 21 enhances cell adhesion and migration by inducing integrin trafficking. Rab21 is present in early endosomes; it functions in the endocytosis of integrins, cell-extracellular matrix adhesion, and motility [52]. In its active form, Rab 21 associates more strongly with beta1 integrin in endocytic vesicles and thus, nucleotide exchange is important in endocytosis. Transport by microtubule- or actin-based motor proteins facilitates timely orientation of vesicles for fusion *in vivo*. Rab-GTPases are perfect factors to regulate the movement of vesicles by controlling the motor protein recruitment of specific target membranes [56].

Carcinoma-associated fibroblasts (CAFs) can promote the progression of tumors in different ways, generating a situation that enables the invasion of cancer cells through extracellular remodeling. Rab21 is involved in promoting cancer cell invasion promoted by CAFs. Rab21 is required for CAFs to promote invasion by cancer cells and facilitates integrin α5 accumulation for force-mediated matrix remodeling at the plasma membrane [57].

Tumor cell invasion and metastasis is increased by hypoxia, which modifies Rab11, a vital molecule for vesicular trafficking through maintenance of microtubule stability. Hypoxia increases integrin α6β4 expression; this increase depends on Rab11 [58]. The oncogene R-Ras, which enhances integrin-mediated cell adhesion and spreading, localizes at the leading edge of migrating cells to regulate membrane protrusion. R-Ras undergoes endocytosis by various pathways, one of which is mediated by vesicles of Rab11. Constitutively, active R-Ras increases the number of Rab11 vesicles. R-Ras is also colocalized with β1-integrin [59]. Thus, integrins appear to have a major part in regulation that occurs between R-Ras and Rab11.

α5β1 integrin cycles between an intracellular Rab 25 compartment and the pseudopodial tip region of migrating cells, where Rab 25 is also localized. Chia and Tang have reported that Rab25 is important in epithelial tumor progression and aggressiveness, associating with α5β1 integrin to increase tumor cell invasion. Rab25 associates with α5β1 integrin to enhance migration and invasion of cells in three-dimensional microenvironments, and directs its recycling to dynamic ruffling protrusions at the leading edge of migrating cells [60]. Rab25 acts directly or through its effector, Rab-coupling protein, to hold together integrin and epidermal growth factor receptor. Rab25, which induces both oncogenic recycling and signaling of the integrin and epidermal growth-factor receptor [25,53] also controls α5β1 integrin at the leading edge, thereby promoting an invasive mode of migration in three-dimensional matrices. Such invasion is mainly dependent on fibronectin, α5β1 integrin, and the interaction of Rab25 with β1 integrin [61]. The association of Rab25 and α5β1 integrin causes enhanced localization of vesicles that deliver integrin to the plasma membrane [53]. In human colorectal adenocarcinomas, Rab25 functions as a tumor suppressor at independent cancer stages by regulating protein trafficking to cell surfaces [25].

The VEGF receptor, VEGFR, is one of the most important positive regulators of new blood vessel growth. Its downstream signaling events have been well studied. Activation of VEGFR1 enhances a Rab4A-dependent pathway that transports αvβ3-integrin from early endosomes to the plasma membrane. This signaling pathway is associated with VEGF-driven fibronectin polymerization in endothelial cells. Rab4a and the integrin αvβ3 are involved in VEGFR1-mediated promotion of endothelial tubule branching in an organotypic model of angiogenesis [62].

The RAB-coupling protein (RAB11FIP1) is a driving force for the 8p11-12 amplicon in human breast cancer and mouse xenograft models of mammary carcinogenesis. One study showed that α5β1 associates with RAB11FIP1 at the tips of pseudopods, causing increased recycling of α5β1 integrin[63]. Since Rab 25 binds to Rab11 FIP1/RCP, it is hypothesized that interaction between them may control α5β1 trafficking. In summary all these reports suggest that Rab GTPases play an important role in integrin trafficking that regulates membrane protrusion activity, which in turn aid in cancer cell migration and invasion.

## Conclusion

The involvement of Rab GTPases in regulating membrane trafficking between organelles occurs by recruitment of effector proteins. Immunodeficiencies, cancer, and neurological disorders are associated with functional impairments of Rab signaling pathways [12]. Alteration or mutation of Rab proteins and/or their effectors may cause many human diseases, including cancer.

Alterations in Rab25, Rab7, Rab5, and Rab11 have been reported to cause different types of cancers. Neuronal dysfunction is caused by abnormalities in Rab1 and Rab7; retinal degeneration is caused by abnormalities in Rab8; and both immune and pigmentation disorders are caused by abnormalities in Rab27 and Rab38 [18,64-66]. Hence, Rab GTPases could serve as primary drug targets. Their interactive molecules may provide pathways for novel therapeutic interventions.

## Competing interests

The authors declare that they have no competing interests.

## Authors' contributions

DS wrote the first draft of the review. SA finalized the draft and figures. All authors read and approved the final manuscript.

## Supplementary Material

Additional file 1 **A. File showing various types of Rab GTPases and their localization**. Detailed references are given in the additional file B. Click here for file
