# Supplementary material for: Integrin-mediated function of Rab GTPases in cancer progression
Source: Mol Cancer. 2010 Dec 9;9:312. doi: 10.1186/1476-4598-9-312 (PMC3003658; doi:10.1186/1476-4598-9-312)
Supplement: Additional file 1 — A. File showing various types of Rab GTPases and their localization. Detailed references are given in the additional file B. [file 1476-4598-9-312-S1.PDF]

1 A

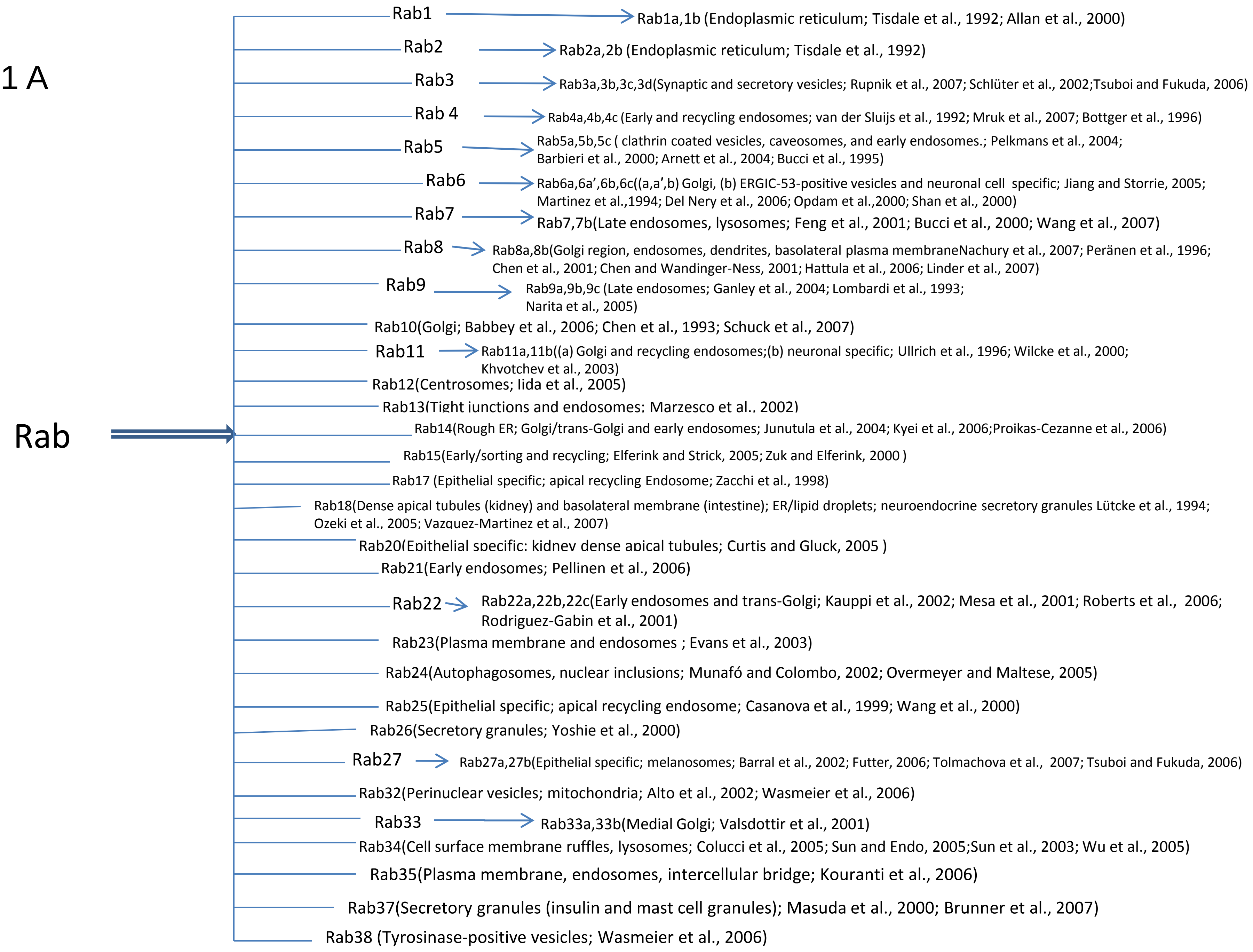
Rab
Rab1
Rab1a,1b (Endoplasmic reticulum; Tisdale et al., 1992; Allan et al., 2000)
Rab2
Rab2a,2b (Endoplasmic reticulum; Tisdale et al., 1992)
Rab3
Rab3a,3b,3c,3d(Synaptic and secretory vesicles; Rupnik et al., 2007; Schlüter et al., 2002;Tsuboi and Fukuda, 2006)
Rab 4
Rab4a,4b,4c (Early and recycling endosomes; van der Sluijs et al., 1992; Mruk et al., 2007; Bottger et al., 1996)
Rab5
Rab5a,5b,5c (clathrin coated vesicles, caveosomes, and early endosomes.; Pelkmans et al., 2004; Barbieri et al., 2000; Arnett et al., 2004; Bucci et al., 1995)
Rab6
Rab6a,6a',6b,6c((a,a',b) Golgi, (b) ERGIC-53-positive vesicles and neuronal cell specific; Jiang and Storrie, 2005; Martinez et al.,1994; Del Nery et al., 2006; Opdam et al.,2000; Shan et al., 2000)
Rab7
Rab7,7b(Late endosomes, lysosomes; Feng et al., 2001; Bucci et al., 2000; Wang et al., 2007)
Rab8
Rab8a,8b(Golgi region, endosomes, dendrites, basolateral plasma membraneNachury et al., 2007; Peränen et al., 1996; Chen et al., 2001; Chen and Wandinger-Ness, 2001; Hattula et al., 2006; Linder et al., 2007)
Rab9
Rab9a,9b,9c (Late endosomes; Ganley et al., 2004; Lombardi et al., 1993; Narita et al., 2005)
Rab10(Golgi; Babbey et al., 2006; Chen et al., 1993; Schuck et al., 2007)
Rab11
Rab11a,11b((a) Golgi and recycling endosomes;(b) neuronal specific; Ullrich et al., 1996; Wilcke et al., 2000; Khvotchev et al., 2003)
Rab12(Centrosomes; Iida et al., 2005)
Rab13(Tight junctions and endosomes: Marzesco et al.. 2002)
Rab14(Rough ER; Golgi/trans-Golgi and early endosomes; Junutula et al., 2004; Kyei et al., 2006;Proikas-Cezanne et al., 2006)
Rab15(Early/sorting and recycling; Elferink and Strick, 2005; Zuk and Elferink, 2000)
Rab17 (Epithelial specific; apical recycling Endosome; Zacchi et al., 1998)
Rab18(Dense apical tubules (kidney) and basolateral membrane (intestine); ER/lipid droplets; neuroendocrine secretory granules Lütcke et al., 1994; Ozeki et al., 2005; Vazquez-Martinez et al., 2007)
Rab20(Epithelial specific: kidney dense apical tubules; Curtis and Gluck, 2005)
Rab21(Early endosomes; Pellinen et al., 2006)
Rab22
Rab22a,22b,22c(Early endosomes and trans-Golgi; Kauppi et al., 2002; Mesa et al., 2001; Roberts et al., 2006; Rodriguez-Gabin et al., 2001)
Rab23(Plasma membrane and endosomes ; Evans et al., 2003)
Rab24(Autophagosomes, nuclear inclusions; Munafó and Colombo, 2002; Overmeyer and Maltese, 2005)
Rab25(Epithelial specific; apical recycling endosome; Casanova et al., 1999; Wang et al., 2000)
Rab26(Secretory granules; Yoshie et al., 2000)
Rab27
Rab27a,27b(Epithelial specific; melanosomes; Barral et al., 2002; Futter, 2006; Tolmachova et al., 2007; Tsuboi and Fukuda, 2006)
Rab32(Perinuclear vesicles; mitochondria; Alto et al., 2002; Wasmeier et al., 2006)
Rab33
Rab33a,33b(Medial Golgi; Valsdottir et al., 2001)
Rab34(Cell surface membrane ruffles, lysosomes; Colucci et al., 2005; Sun and Endo, 2005;Sun et al., 2003; Wu et al., 2005)
Rab35(Plasma membrane, endosomes, intercellular bridge; Kouranti et al., 2006)
Rab37(Secretory granules (insulin and mast cell granules); Masuda et al., 2000; Brunner et al., 2007)
Rab38 (Tyrosinase-positive vesicles; Wasmeier et al., 2006)

1B

Tisdale, E. J., Bourne, J. R., Khosravi-Far, R., Der, C. J. and Balch, W. E. (1992). GTP-binding mutants of rab1 and rab2 are potent inhibitors of vesicular transport from the endoplasmic reticulum to the Golgi complex. *J. Cell Biol.* **119**, 749-761

Allan, B. B., Moyer, B. D. and Balch, W. E. (2000). Rab1 recruitment of p115 into a cis-SNARE complex: programming budding COPII vesicles for fusion. *Science* **289**, 444-448.

Rupnik, M., Kreft, M., Nothias, F., Grilc, S., Bobanovic, L. K., Johannes, L., Kiauta, T., Vernier, P., Darchen, F. and Zorec, R. (2007). Distinct role of Rab3A and Rab3B in secretory activity of rat melanotrophs. *Am. J. Physiol. Cell Physiol.* **292**, C98-C105

Schlüter, O. M., Khvotchev, M., Jahn, R. and Sudhof, T. C. (2002). Localization versus function of Rab3 proteins. Evidence for a common regulatory role in controlling fusion. *J. Biol. Chem.* **277**, 40919-40929

Tsuboi, T. and Fukuda, M. (2006). Rab3A and Rab27A cooperatively regulate the docking step of dense-core vesicle exocytosis in PC12 cells. *J. Cell Sci.* **119**, 2196-2203.

van der Sluijs, P., Hull, M., Webster, P., Male, P., Goud, B. and Mellman, I. (1992). The small GTP-binding protein rab4 controls an early sorting event on the endocytic pathway. *Cell* **70**, 729-740

Mruk, D. D., Lau, A. S., Sarkar, O. and Xia, W. (2007). Rab4A GTPase–catenin interactions are involved in cell junction dynamics in the testis. *J. Androl.* **28**, 742-754.

Bottger, G., Nagelkerken, B. and van der Sluijs, P. (1996). Rab4 and Rab7 define distinct nonoverlapping endosomal compartments. *J. Biol. Chem.* **271**, 29191-29197.

Pelkmans, L., Burli, T., Zerial, M. and Helenius, A. (2004). Caveolin-stabilized membrane domains as multifunctional transport and sorting devices in endocytic membrane traffic. *Cell* **118**, 767-780

Barbieri, M. A., Roberts, R. L., Gumusboga, A., Highfield, H., Alvarez-Dominguez, C., Wells, A. and Stahl, P. D. (2000). Epidermal growth factor and membrane trafficking. EGF receptor activation of endocytosis requires Rab5a. *J. Cell Biol.* **151**, 539-550

Arnett, A. L., Bayazitov, I., Blaabjerg, M., Fang, L., Zimmer, J. and Baskys, A. (2004). Antisense oligonucleotide against GTPase Rab5b inhibits metabotropic agonist DHPG-induced neuroprotection. *Brain Res.* **1028**, 59-65

Bucci, C., Lutcke, A., Steele-Mortimer, O., Olkkonen, V. M., Dupree, P., Chiariello, M., Bruni, C. B., Simons, K. and Zerial, M. (1995). Co-operative regulation of endocytosis by three Rab5 isoforms. *FEBS Lett.* **366**, 65-71

Jiang, S. and Storrie, B. (2005). Cisternal rab proteins regulate Golgi apparatus redistribution in response to hypotonic stress. *Mol. Biol. Cell* **16**, 2586-2596

Martinez, O., Schmidt, A., Salamero, J., Hoflack, B., Roa, M. and Goud, B. (1994). The small GTP-binding protein rab6 functions in intra-Golgi transport. *J. Cell Biol.* **127**, 1575-1588

Del Nery, E., Miserey-Lenkei, S., Falguieres, T., Nizak, C., Johannes, L., Perez, F. and Goud, B. (2006). Rab6A and Rab6A' GTPases play non-overlapping roles in membrane trafficking. *Traffic* **7**, 394-407

Opdam, F. J., Echard, A., Croes, H. J., van den Hurk, J. A., van de Vorstenbosch, R. A., Ginsel, L. A., Goud, B. and Fransen, J. A. (2000). The small GTPase Rab6B, a novel Rab6 subfamily member, is cell-type specifically expressed and localised to the Golgi apparatus. *J. Cell Sci.* **113**, 2725-2735

Shan, J., Mason, J. M., Yuan, L., Barcia, M., Porti, D., Calabro, A., Budman, D., Vinciguerra, V. and Xu, H. (2000). Rab6c, a new member of the rab gene family, is involved in drug resistance in MCF7/AdrR cells. *Gene* **257**, 67-75

Feng, Y., Press, B., Chen, W., Zimmerman, J. and Wandinger-Ness, A. (2001). Expression and properties of Rab7 in endosome function. *Meth. Enzymol.* **329**, 175-187.

Bucci, C., Thomsen, P., Nicoziani, P., McCarthy, J. and van Deurs, B. (2000). Rab7: a key to lysosome biogenesis. *Mol. Biol. Cell* **11**, 467-480.

Wang, Y., Chen, T., Han, C., He, D., Liu, H., An, H., Cai, Z. and Cao, X. (2007). Lysosome-associated small Rab GTPase Rab7b negatively regulates TLR4 signaling in macrophages by promoting lysosomal degradation of TLR4. *Blood* **110**, 962-971

Nachury, M. V., Loktev, A. V., Zhang, Q., Westlake, C. J., Peranen, J., Merdes, A., Slusarski, D. C., Scheller, R. H., Bazan, J. F., Sheffield, V. C. et al. (2007). A core complex of BBS proteins cooperates with the GTPase Rab8 to promote ciliary membrane biogenesis. *Cell* **129**, 1201-1213

Peränen, J., Auvinen, P., Virta, H., Wepf, R. and Simons, K. (1996). Rab8 promotes polarized membrane transport through reorganization of actin and microtubules in fibroblasts. *J. Cell Biol.* **135**, 153-167

Chen, S., Liang, M. C., Chia, J. N., Ngsee, J. K. and Ting, A. E. (2001). Rab8b and its interacting partner TRIP8b are involved in regulated secretion in AtT20 cells. *J. Biol. Chem.* **276**, 13209-13216.

Chen, W. and Wandinger-Ness, A. (2001). Expression and functional analyses of Rab8 and Rab11a in exocytic transport from trans-Golgi network. *Meth. Enzymol.* **329**, 165-175.

Hattula, K., Furuhjelm, J., Tikkanen, J., Tanhuanpaa, K., Laakkonen, P. and Peranen, J. (2006). Characterization of the Rab8-specific membrane traffic route linked to protrusion formation. *J. Cell Sci.* **119**, 4866-4877

Linder, M. D., Uronen, R. L., Holtta-Vuori, M., van der Sluijs, P., Peranen, J. and Ikonen, E. (2007). Rab8-dependent recycling promotes endosomal cholesterol removal in normal and sphingolipidosis cells. *Mol. Biol. Cell* **18**, 47-56

Ganley, I. G., Carroll, K., Bittova, L. and Pfeffer, S. (2004). Rab9 GTPase regulates late endosome size and requires effector interaction for its stability. *Mol. Biol. Cell* **15**, 5420-5430

Lombardi, D., Soldati, T., Riederer, M. A., Goda, Y., Zerial, M. and Pfeffer, S. R. (1993). Rab9 functions in transport between late endosomes and the trans Golgi network. *EMBO J.* **12**, 677-682

Narita, K., Choudhury, A., Dobrenis, K., Sharma, D. K., Holicky, E. L., Marks, D. L., Walkley, S. U. and Pagano, R. E. (2005). Protein transduction of Rab9 in Niemann-Pick C cells reduces cholesterol storage. *FASEB J.* **19**, 1558-1560

Babbey, C. M., Ahktar, N., Wang, E., Chen, C. C., Grant, B. D. and Dunn, K. W. (2006). Rab10 regulates membrane transport through early endosomes of polarized Madin-Darby canine kidney cells. *Mol. Biol. Cell* **17**, 3156-3175

Chen, Y. T., Holcomb, C. and Moore, H. P. (1993). Expression and localization of two low molecular weight GTP-binding proteins, Rab8 and Rab10, by epitope tag. *Proc. Natl. Acad. Sci. USA* **90**, 6508-6512

Schuck, S., Gerl, M. J., Ang, A., Manninen, A., Keller, P., Mellman, I. and Simons, K. (2007). Rab10 is involved in basolateral transport in polarized Madin-Darby canine kidney cells. *Traffic* **8**, 47-60

Ullrich, O., Reinsch, S., Urbe, S., Zerial, M. and Parton, R. G. (1996). Rab11 regulates recycling through the pericentriolar recycling endosome. *J. Cell Biol.* **135**, 913-924

Wilcke, M., Johannes, L., Galli, T., Mayau, V., Goud, B. and Salamero, J. (2000). Rab11 regulates the compartmentalization of early endosomes required for efficient transport from early endosomes to the trans-golgi network. *J. Cell Biol.* **151**, 1207-1220

Khvochev, M. V., Ren. M., Takamori, S., Jahn, R. and Sudhof, T. C. (2003). Divergent functions of neuronal Rab11b in Ca2+ regulated versus constituative exocytosis. *J. Neurosci.* **23**, 10531-10539

Iida, H., Noda, M., Kaneko, T., Doiguchi, M. and Mori, T. (2005). Identification of rab12 as a vesicle-associated small GTPase highly expressed in Sertoli cells of rat testis. *Mol. Reprod. Dev.* **71**, 178-185

Marzesco, A. M., Dunia, I., Pandjaitan, R., Recouvreur, M., Dauzonne, D., Benedetti, E. L., Louvard, D. and Zahraoui, A. (2002). The small GTPase Rab13 regulates assembly of functional tight junctions in epithelial cells. *Mol. Biol. Cell* **13**, 1819-1831

Junutula, J. R., De Maziere, A. M., Peden, A. A., Ervin, K. E., Advani, R. J., van Dijk, S. M., Klumperman, J. and Scheller, R. H. (2004). Rab14 is involved in membrane trafficking between the Golgi complex and endosomes. *Mol. Biol. Cell* **15**, 2218-2229

Proikas-Cezanne, T., Gaugel, A., Frickey, T. and Nordheim, A. (2006). Rab14 is part of the early endosomal clathrin-coated TGN microdomain. *FEBS Lett.* **580**, 5241-5246.

Elferink, L. A. and Strick, D. J. (2005). Functional properties of rab15 effector protein in endocytic recycling. *Meth. Enzymol.* **403**, 732-743

Zuk, P. A. and Elferink, L. A. (2000). Rab15 differentially regulates early endocytic trafficking. *J. Biol. Chem.* **275**, 26754-26764.

Zacchi, P., Stenmark, H., Parton, R. G., Orioli, D., Lim, F., Giner, A., Mellman, I., Zerial, M. and Murphy, C. (1998). Rab17 regulates membrane trafficking through apical recycling endosomes in polarized epithelial cells. *J. Cell Biol.* **140**, 1039-1053

Lütcke, A., Parton, R. G., Murphy, C., Olkkonen, V. M., Dupree, P., Valencia, A., Simons, K. and Zerial, M. (1994). Cloning and subcellular localization of novel rab proteins reveals polarized and cell type-specific expression. *J. Cell Sci.* **107**, 3437-3448

Ozeki, S., Cheng, J., Tauchi-Sato, K., Hatano, N., Taniguchi, H. and Fujimoto, T. (2005). Rab18 localizes to lipid droplets and induces their close apposition to the endoplasmic reticulum-derived membrane. *J. Cell Sci.* **118**, 2601-2611

Vazquez-Martinez, R., Cruz-Garcia, D., Duran-Prado, M., Peinado, J. R., Castano, J. P. and Malagon, M. M. (2007). Rab18 inhibits secretory activity in neuroendocrine cells by interacting with secretory granules. *Traffic* **8**, 867-882

Curtis, L. M. and Gluck, S. (2005). Distribution of Rab GTPases in mouse kidney and comparison with vacuolar H+-ATPase. *Nephron Physiol.* **100**, p31-p42

Pellinen, T., Arjonen, A., Vuoriluoto, K., Kallio, K., Fransen, J. A. and Ivaska, J. (2006). Small GTPase Rab21 regulates cell adhesion and controls endosomal traffic of beta1-integrins. *J. Cell Biol.* **173**, 767-780

Kauppi, M., Simonsen, A., Bremnes, B., Vieira, A., Callaghan, J., Stenmark, H. and Olkkonen, V. M. (2002). The small GTPase Rab22 interacts with EEA1 and controls endosomal membrane trafficking. *J. Cell Sci.* **115**, 899-911

Mesa, R., Salomon, C., Roggero, M., Stahl, P. D. and Mayorga, L. S. (2001). Rab22a affects the morphology and function of the endocytic pathway. *J. Cell Sci.* **114**, 4041-4049

Roberts, E. A., Chua, J., Kyei, G. B. and Deretic, V. (2006). Higher order Rab programming in phagolysosome biogenesis. *J. Cell Biol.* **174**, 923-929

Rodriguez-Gabin, A. G., Cammer, M., Almazan, G., Charron, M. and Larocca, J. N. (2001). Role of rRAB22b, an oligodendrocyte protein, in regulation of transport of vesicles from trans Golgi to endocytic compartments. *J. Neurosci. Res.* **66**, 1149-1160

Evans, T. M., Ferguson, C., Wainwright, B. J., Parton, R. G. and Wicking, C. (2003). Rab23, a negative regulator of hedgehog signaling, localizes to the plasma membrane and the endocytic pathway. *Traffic* **4**, 869-884

Munafó, D. B. and Colombo, M. I. (2002). Induction of autophagy causes dramatic changes in the subcellular distribution of GFP-Rab24. *Traffic* **3**, 472-482

Overmeyer, J. H. and Maltese, W. A. (2005). Tyrosine phosphorylation of Rab proteins. *Meth. Enzymol.* **403**, 194-202

Casanova, J. E., Wang, X., Kumar, R., Bhartur, S. G., Navarre, J., Woodrum, J. E., Altschuler, Y., Ray, G. S. and Goldenring, J. R. (1999). Association of Rab25 and Rab11a with the apical recycling system of polarized Madin-Darby canine kidney cells. *Mol. Biol. Cell* **10**, 47-61.

Wang, X., Kumar, R., Navarre, J., Casanova, J. E. and Goldenring, J. R. (2000). Regulation of vesicle trafficking in madin-darby canine kidney cells by Rab11a and Rab25. *J. Biol. Chem.* **275**, 29138-29146

Yoshie, S., Imai, A., Nashida, T. and Shimomura, H. (2000). Expression, characterization, and localization of Rab26, a low molecular weight GTP-binding protein, in the rat parotid gland. *Histochem. Cell Biol.* **113**, 259-263

Barral, D. C., Ramalho, J. S., Anders, R., Hume, A. N., Knapton, H. J., Tolmachova, T., Collinson, L. M., Goulding, D., Authi, K. S. and Seabra, M. C. (2002). Functional redundancy of Rab27 proteins and the pathogenesis of Griscelli syndrome. *J. Clin. Invest.* **110**, 247-257

Futter, C. E. (2006). The molecular regulation of organelle transport in mammalian retinal pigment epithelial cells. *Pigment Cell Res.* **19**, 104-111

Tolmachova, T., Abrink, M., Futter, C. E., Authi, K. S. and Seabra, M. C. (2007). Rab27b regulates number and secretion of platelet dense granules. *Proc. Natl. Acad. Sci. USA* **104**, 5872-5877.

Alto, N. M., Soderling, J. and Scott, J. D. (2002). Rab32 is an A-kinase anchoring protein and participates in mitochondrial dynamics. *J. Cell Biol.* **158**, 659-668

Wasmeier, C., Romao, M., Plowright, L., Bennett, D. C., Raposo, G. and Seabra, M. C. (2006). Rab38 and Rab32 control post-Golgi trafficking of melanogenic enzymes. *J. Cell Biol.* **175**, 271-281

Valsdottir, R., Hashimoto, H., Ashman, K., Koda, T., Storrie, B. and Nilsson, T. (2001). Identification of rabaptin-5, rabex-5, and GM130 as putative effectors of rab33b, a regulator of retrograde traffic between the Golgi apparatus and ER. *FEBS Lett.* **508**, 201-209

Colucci, A. M., Spinosa, M. R. and Bucci, C. (2005). Expression, assay, and functional properties of RILP. *Meth. Enzymol.* **403**, 664-675

Sun, P. and Endo, T. (2005). Assays for functional properties of Rab34 in macropinosome formation. *Meth. Enzymol.* **403**, 229-243

Sun, P., Yamamoto, H., Suetsugu, S., Miki, H., Takenawa, T. and Endo, T. (2003). Small GTPase Rah/Rab34 is associated with membrane ruffles and macropinosomes and promotes macropinosome formation. *J. Biol. Chem.* **278**, 4063-4071

Wu, M., Wang, T., Loh, E., Hong, W. and Song, H. (2005). Structural basis for recruitment of RILP by small GTPase Rab7. *EMBO J.* **24**, 1491-1501

Kouranti, I., Sachse, M., Arouche, N., Goud, B. and Echard, A. (2006). Rab35 regulates an endocytic recycling pathway essential for the terminal steps of cytokinesis. *Curr. Biol.* **16**, 1719-1725

Masuda, E. S., Luo, Y., Young, C., Shen, M., Rossi, A. B., Huang, B. C., Yu, S., Bennett, M. K., Payan, D. G. and Scheller, R. H. (2000). Rab37 is a novel mast cell specific GTPase localized to secretory granules. *FEBS Lett.* **470**, 61-64

Brunner, Y., Coute, Y., Iezzi, M., Foti, M., Fukuda, M., Hochstrasser, D., Wollheim, C. and Sanchez, J. C. (2007). Proteomic analysis of insulin secretory granules. *Mol. Cell. Proteomics* **6**, 1007-1017
